# Evaluation of the impact of bundled nursing strategies on the prevention of venous thromboembolism in patients with cerebral hemorrhage

**DOI:** 10.1097/MD.0000000000038725

**Published:** 2024-07-12

**Authors:** Lu Hongfang, Tian Yangyang, Zhao Lijuan, Sun Na

**Affiliations:** a Neurosurgery, Baoji People’s Hospital, Baoji, Shaanxi, China; b Department of Nephrology, Baoji People’s Hospital, Baoji, Shaanxi, China.

**Keywords:** bundled care, cerebral hemorrhage, intervention, thrombus, venous blood, VTE

## Abstract

To investigate the effectiveness and value of bundled nursing strategies for venous thromboembolism prevention in nonsurgical patients with cerebral hemorrhage, 200 patients who underwent treatment for cerebral hemorrhage in our hospital from January 2023 to July 2023 were chosen as the study subjects. Patients were divided into control group and experimental group according to different treatment methods. For patients in the control group, regular care was used, while for patients in the observation group, bundled care was used for intervention. This study used a venous thromboembolism risk factor assessment form to assess the probability of patients suffering from venous thromboembolism. It used the incidence of venous thromboembolism, disease cognition level, coagulation function, fibrinolysis, changes in blood routine, exercise ability, improvement in quality of life, and patient satisfaction with nursing mode as detection indicators to obtain the changes in various indicator values and patient satisfaction scores under different nursing interventions. Prior to nursing intervention, the significant statistical differences did not exist (*P* > .05) between the control group and the observation group in terms of general information, number of venous thromboembolism risk levels, degree of disease cognition, coagulation function, blood routine, exercise ability, quality of life, and fibrinolysis indicators. After a period of nursing intervention, the significant statistical difference existed (*P* < .05) between the control group and the observation group in terms of general information, number of venous thromboembolism risk levels, degree of disease cognition, coagulation function, blood routine, exercise ability, quality of life, and fibrinolysis indicators. In the patient satisfaction score, the satisfaction scores of male and female patients with general care were 0.865 and 0.878, respectively, and the satisfaction scores for bundled care were 0.942 and 0.965, respectively. In conclusion, bundled care can better improve the coagulation status and blood routine indexes of nonsurgical patients with cerebral hemorrhage compared with ordinary care, thus contributing to better prevention of venous thromboembolism in nonsurgical patients with cerebral hemorrhage.

## 1. Introduction

In the modern medical system, with the continuous optimization of treatment methods for cerebral hemorrhage and the improvement of survival rate, the management of long-term complications has become particularly important.^[1,2]^ A common complication in the rehabilitation of nonsurgical patients with cerebral hemorrhage is venous thromboembolism (VTE), which is a serious circulatory system disease that includes deep vein thrombosis and pulmonary embolism.^[3,4]^ It is estimated that about 20% to 40% of nonsurgical patients with unprotected cerebral hemorrhage will develop VTE, and this proportion will significantly increase without appropriate preventive measures.^[5]^ VTE after cerebral hemorrhage grows the risk of death for patients and leads to long-term disability, increased hospitalization time, and medical costs. Due to limited movement in nonsurgical patients with cerebral hemorrhage, their venous blood flow slows down, which, combined with endothelial damage and hypercoagulable blood, greatly increases the risk of thrombosis.^[6,7]^ In addition, the patient’s age, past medical history, and individual physiological reactions can also affect the incidence of VTE.^[8]^ In recent years, the bundled nursing strategy, as a multi-dimensional and comprehensive nursing method, has received increasing attention from clinical research.^[9]^ The bundled nursing strategy is a method that integrates multiple prevention and treatment measures, and improves nursing effectiveness by coordinating and optimizing various nursing methods.^[10]^ In the prevention of VTE in nonsurgical patients with cerebral hemorrhage, this strategy usually includes multiple aspects such as drug therapy, physical therapy, nursing interventions, and lifestyle adjustments. Drug therapy mainly involves the use of anticoagulants to reduce blood clotting, while physical therapy includes wearing elastic socks and regularly engaging in lower limb activities.^[11]^ In addition, nursing interventions such as regular flipping, massage, and skin care, as well as encouraging patients to engage in appropriate physical activity, are important measures to prevent VTE. However, although bundled nursing strategies have theoretical advantages, their effectiveness in practical applications still needs to be systematically evaluated and analyzed.^[12]^ On the grounds of this background, this study is for observing the incidence of VTE in nonsurgical patients with cerebral hemorrhage after applying a bundled nursing strategy, and evaluate the effectiveness and feasibility of this strategy in practical clinical nursing. This study not only helps to enhance understanding of VTE prevention in nonsurgical patients with cerebral hemorrhage, but also provides more effective nursing plans for clinical practice. A systematic evaluation of bundled care strategies can help healthcare professionals better understand the interplay of various preventive measures and their impact on patient prognosis. This can provide scientific basis for clinical nursing practice.

## 2. Materials and methods

### 2.1. General information

A total of 200 patients who underwent cerebral hemorrhage treatment in our hospital from January 2023 to July 2023 were selected as the study subjects, all 200 patients underwent nonsurgical treatment for cerebral hemorrhage in our hospital and met the following inclusion criteria. Two hundred patients were divided into 2 groups according to different treatment methods, namely the control group (CG) (n1 = 100) and the observation group (OG) (n2 = 100). The inclusion criteria for patients include: ① The patient was clearly confirmed by imaging diagnosis before treatment. ② No mental abnormalities, able to communicate normally, with autonomous ability. ③ The patient suffered from severe limb movement limitation or fell into a coma after treatment. ④ No abnormal coagulation function. ⑤ No severe organ dysfunction such as heart, lungs, and brain. ⑥ With the consent of myself and my family, I am able to sign an informed consent form. The exclusion criteria for patients include: ① patients with lower limb vascular disease. ② Patients with pelvic or leg fractures. ③ Have a past medical history of cerebral infarction attacks. ④ Patients who have undergone stent implantation surgery or are currently taking anticoagulant drugs. ⑤ Family members unable to complete the questionnaire. ⑥ Patients with abnormally high muscle tone. ⑦ Patients who are unable to sign informed consent forms and participate in clinical studies. This study has been approved by Baoji People’s Hospital ethics committee.

### 2.2. Outcome measures

The observation indicators of this study include the incidence of VTE, disease cognitive level, coagulation function, fibrinolysis, blood routine changes, lower limb motor (LLM) function, functional walking ability (WA), improvement in quality of life (LQ), and patient satisfaction with the nursing model.

### 2.3. Research method

This study implemented standardized care for CG patients. The content of standard nursing includes creating a comfortable hospitalization environment, maintaining appropriate temperature and humidity, and guiding family members to maintain quiet during visits. In addition, nursing staff need to regularly check the patient’s lower limbs, record skin color and temperature, inquire about discomfort such as swelling or pain, and promote VTE knowledge to the patient and their family. The patient’s diet is mainly light and high protein, encouraging them to eat more fresh fruits and vegetables to prevent constipation, and guiding them to exercise passively and actively. In addition, patients in the control group needed to start rehabilitation of basic passive movements 2 weeks after nonsurgical treatment of cerebral hemorrhage, but not systematic rehabilitation.

In addition to receiving routine care, the OG patients also received comprehensive bundled and early warning nursing interventions. Specific measures include VTE risk assessment, psychological support, health education, disease monitoring, rehabilitation training, and intravenous care. The VTE risk factor assessment form is showcased in Table 1.

**Table 1:** VTE risk factor assessment table.

Risk factor-related indicators	Score
Stroke	5
Multiple trauma	5
Paralysis	5
Medical patients undergoing major surgery	4
Severe infection after major surgery	4
Hypercoagulability	3
History of deep vein thrombosis and pulmonary embolism	3
Over 60 years of age	3
Bedridden for more than 3 days	2
Surgery lasting more than 1 hour	2
Use of central venous catheter	2
Have a malignant tumor	2
Obesity	2
Pregnant or within 3 months after delivery	1
Major surgery within the last 1 month	1
Over 40 years of age but under 60 years of age	1

Table 1 presents the relevant influencing factors and their scores for VTE risk assessment. The VTE risk assessment specifically uses the Caprini Deep Venous Thrombosis Risk Assessment Form, which divides patients into low-risk (1–2 points), medium risk (3–4 points), and high-risk (5 points) based on factors such as bed rest time, age, and medical condition. Psychological support refers to communicating with patients and their families, explaining precautions, complications, prevention measures, and reducing psychological pressure on patients and their families. Health education refers to various forms of health education provided by a team of experts, including oral, written, face-to-face demonstrations, and multimedia. Disease monitoring refers to the evaluation of VTE risk factors twice a week, regular testing of coagulation function and D-dimer (D-D) levels for patients with moderate to high risk, monitoring of symptoms such as chest and back pain, chest tightness, syncope, and hemoptysis, and timely follow-up of lower limb ultrasound. Early rehabilitation training refers to starting early training within 48 hours to 2 weeks for stable patients with moderate to high risk, guiding family members to assist patients in early passive exercise and gradually increasing their physical activity. It uses an air wave pressure circulation therapy device, a medium frequency pulse electric therapy device, and a lower limb power car to promote limb activity and blood circulation. Venous care refers to the use of 0.9% sodium chloride injection for pulsed venous catheterization, improving puncture techniques and reducing repeated punctures of the same vein. When puncturing, priority should be given to venipuncture on the healthy side of the upper limb, and the use time of the indwelling needle should be limited. It selects PICC catheterization or subclavian deep vein catheterization according to the patient’s condition, reduces the number of venous punctures, and applies Hirudoid topically at the catheterization site to prevent phlebitis.

### 2.4. Data processing tools

This study uses SPSS 23.0 to statistically analyze all the data generated during the experimental process. This study uses (mean ± standard deviation) to represent econometric data, and *t*-tests are used for inter group comparisons. Count data is represented using frequency ± percentage, and chi square test is used for count data. *P* < .05 demonstrates significant statistical significance (SS) in the comparison results.

## 3. Results

### 3.1. Comparison results of general information

By comparing general indicators such as age, gender, and weight, the comparison results of general information among the participants are showcased in Table 2.

**Table 2:** Comparison of general information.

Program		Control group (n1, %)	Observation group (n2, %)	*χ* ^2^	*P*
Sex	Men	(58, 58%)	(36, 36%)	0.021	.998
	Women	(42, 42%)	(64, 64%)		
Age	Below 40	(22, 22%)	(12, 12%)	0.485	.954
	41–60	(62, 62%)	(74, 74%)		
	Above 61	(16, 16%)	(14, 14%)		
Weight	Normal	(92, 92%)	(95, 95%)	0.326	.862
	Obese	(8, 8%)	(5, 5%)		
Bedtime	≤3 days	(68, 68%)	(75, 75%)	0.257	.864
	>3 days	(32, 32%)	(25, 25%)		
VTE risk level	Low risk	(54, 54%)	(60, 60%)	1.648	.325
	Medium risk	(28, 28%)	(25, 25%)		
	High risk	(18, 18%)	(15, 15%)		

In Table 2, the significant statistical difference (SSD) did not exist (*P* > .05) in the comparison of gender, age, weight, bed rest time, and VTE risk level among the participants. This indicates that the general situation of the CG and the OG patients is relatively similar, with good fairness, and can further carry out comparative nursing.

### 3.2. Comparison of VTE indicators among the participants

After a period of nursing intervention, the comparison of the number of patients with different VTE risk levels between the CG and the OG is showcased in Table 3.

**Table 3:** Comparison of the number of people with different VTE risk classes among the participants.

Time	Groups	Number	VTE risk level			Incidence of high-risk VTE (n, %)
			Low risk	Medium risk	High risk	
Pre-intervention	Control group	100	54	28	18	(18, 18%)
	Observation group	100	60	25	15	(15, 15%)
	*χ* ^2^	/	1.648			0.846
	*P*	/	.325			.112
Two weeks of intervention	Control group	100	50	34	16	(16, 16%)
	Observation group	100	82	12	6	(6, 6%)
	*χ* ^2^	/	1.021			0.952
	*P*	/	.015			.021

In Table 3, the SSD did not exist (*P* > .05) in the comparison of the number of VTE risk levels among the participants before intervention. After 2 weeks of intervention, the SSD existed (*P* < .05) in the comparison of the number of VTE risk levels among the participants. After 2 weeks of intervention, the incidence of high-risk VTE in the CG increased from 18% to 16%, and the incidence of high-risk VTE in the OG increased from 15% to 6%.

### 3.3. Comparison of cognitive levels of lesions among the participants

This study collected the dietary, psychological, drug adverse reactions, and exercise status of 2 groups of patients during different nursing interventions. The dietary, psychological, drug response, and exercise changes of patients were unified as the degree of disease cognition of the patients. The comparison results of disease cognition among the participants are showcased in Table 4.

**Table 4:** Comparison results of lesion awareness level among the participants.

Time	Groups	Number	Healthy diet (n, %)	Mental health (n, %)	No adverse drug reactions (n, %)	Active exercise (n, %)
Pre-intervention	Control group	100	(62, 62%)	(52, 52%)	(55, 55%)	(59, 59%)
	Observation group	100	(68, 68%)	(53, 53%)	(51, 51%)	(62, 62%)
	*χ* ^2^	/	1.628			
	*P*	/	.257			
Two weeks of intervention	Control group	100	(65, 65%)	(71, 71%)	(62, 62%)	(69, 69%)
	Observation group	100	(98, 98%)	(99, 99%)	(96, 96%)	(95, 95%)
	*χ* ^2^	/	25.380			
	*P*	/	.012			

* Indicates statistically significant difference between the 2 groups (*P* < .05).

In Table 4, there was no SSD (*P* > .05) in the comparison of lesion cognition among the participants before intervention. After 2 weeks of intervention, there was a SSD (*P* < .05) in the comparison of lesion cognition among the participants.

### 3.4. Comparison of coagulation function among the participants

The activated partial thromboplastin time (APTT), thrombin time (TT), and prothrombin time (PT) were used as coagulation function testing indicators to obtain the changes in coagulation function of the participants before and after different nursing interventions, as indicated in Table 5.

**Table 5:** Comparison of coagulation function of the participants.

Time	Coagulation index	Control group	Observation group	*t*	*P*
Pre-intervention	APTT(s)	26.512 ± 7.234	26.513 ± 7.312	0.023	.956
Two weeks of intervention		31.651 ± 9.285*	42.322 ± 9.596*	2.327	.005
Pre-intervention	TT(s)	25.332 ± 8.212	25.331 ± 8.215	0.158	.894
Two weeks of intervention		26.013 ± 8.135*	29.590 ± 8.052*	3.654	.001
Pre-intervention	PT(s)	15.352 ± 9.110	15.351 ± 9.108	0.064	.963
Two weeks of intervention		15.628 ± 9.654*	19.092 ± 9.575*	2.359	.002

* Indicates statistically significant difference between the 2 groups (*P* < .05).

In Table 5, the APTT, TT, and PT values of the CG and the OG did not possess SS before intervention (*P* > .05). After 2 weeks of intervention, the APTT, TT, and PT values of the OG were higher than those of the CG, and there was a SSD among the participants (*P* < .05).

### 3.5. Comparison of blood routine changes among the participants

Platelet (PLT), white blood cell (WBC), and red blood cell (RBC) were selected as blood routine test indicators. The blood routine comparison results of the 2 groups of patients before and after different nursing interventions are showcased in Table 6.

**Table 6:** Comparison results of blood routine of 2 groups of patients.

Time	Routine blood test	Control group	Observation group	*t*	*P*
Pre-intervention	PLT (×10^9^/L)	226.231 ± 81.602	226.235 ± 81.595	0.005	1.921
Two weeks of intervention		182.356 ± 62.575*	165.391 ± 61.875*	2.264	.018
Pre-intervention	WBC (×10^9^/L)	8.651 ± 2.556	8.654 ± 2.528	0.085	.695
Two weeks of intervention		6.691 ± 1.623*	5.017 ± 1.698*	1.548	.005
Pre-intervention	RBC(×10^12^/L)	4.688 ± 1.522	4.687 ± 1.513	0.063	.582
Two weeks of intervention		4.21 ± 0.891*	4.05 ± 0.840*	1.659	.026

* Indicates statistically significant difference between the 2 groups (*P* < .05).

In Table 6, the PLT, WBC, and RBC values of the CG and OG did not possess SS before intervention (*P* > .05). After 2 weeks of intervention, the PLT, WBC, and RBC values of the OG were below the CG. The PLT, WBC, and RBC values of the CG remained basically unchanged, and there was a SSD between the CG and the OG (*P* < .05).

### 3.6. Comparison results of LLM ability and WA among the participants

The study selected LLM ability and WA as the indicators for motor ability detection, and the comparison results of motor ability among the participants before and after different nursing interventions are showcased in Table 7.

**Table 7:** Comparison results of exercise capacity of 2 groups of patients.

Time	Motor indicators	Control group	Observation group	*t*	*P*
Pre-intervention	Lower extremity mobility	16.482 ± 3.541	16.479 ± 3.545	0.068	.865
Two weeks of intervention		18.651 ± 3.272*	26.125 ± 3.698*	8.236	<.001
Pre-intervention	Walking mobility	2.667 ± 0.782	2.665 ± 0.752	0.395	.758
Two weeks of intervention		2.912 ± 1.021*	4.091 ± 0.959*	3.648	<.001

* Indicates statistically significant difference between the 2 groups (*P* < .05).

In Table 7, there was no significant SS (*P* > .05) in the LLM ability and WA of the CG and OG before intervention, but there was a SSD (*P* < .05) after 2 weeks of intervention.

### 3.7. Comparison of changes in LQ among the participants

The comparison results of the LQ among the participants before and after different nursing interventions are shown in Table 8, by selecting physiological function, physical function, and social function as the testing indicators for LQ.

**Table 8:** Comparative results of the LQ of the participants.

Time	Quality of life indicators	Control group	Observation group	*t*	*P*
Pre-intervention	Physical functioning	63.242 ± 4.585	63.244 ± 4.576	0.085	.954
Two weeks of intervention		68.051 ± 4.024	79.312 ± 3.891	15.621	.001
Pre-intervention	Physical functioning	61.535 ± 3.691	61.533 ± 3.608	0.053	.978
Two weeks of intervention		65.922 ± 4.067*	75.289 ± 4.185*	10.458	<.001
Pre-intervention	Social functioning	62.443 ± 3.653	62.449 ± 3.646	0.047	.982
Two weeks of intervention		69.282 ± 3.850*	78.10 ± 4.11*	12.253	<.001

* Indicates statistically significant difference between the 2 groups (*P* < .05).

In Table 8, there was no significant SS (*P* > .05) in the LLM ability and WA of the CG and OG before intervention, but there was a SSD (*P* < .05) after 2 weeks of intervention.

### 3.8. Comparison of fibrinolysis among the participants

**Figure 1.**
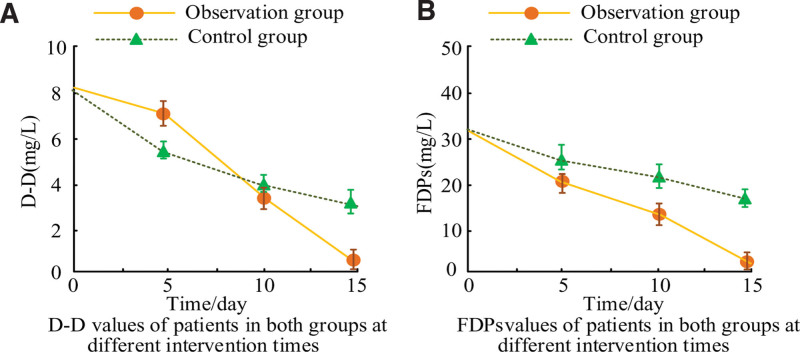
Changes in D-D values and FDPs values in the participants. FDPs = Fibrin Degradation Products.

This study selected D-D and Fibrin Degradation Products (FDPs) as detection indicators for fibrinolysis, and the comparative results of fibrinolysis among the participants are shown in Figure 1.

Figure 1 shows the changes in D-D values and FDPs values of the participants. Figure 1A shows the variation of D-D values of patients in the CG and OG with intervention time. According to Figure 1A, when the intervention time was on the 5th, 10th, and 15th day, the D-D values of the CG patients were 5.482 ± 3.623, 4.024 ± 3.586, and 3.511 ± 3.950, respectively. The D-D values of the OG patients were 7.611 ± 4.125, 3.683 ± 3.742, and 0.755 ± 3.541. Figure 1B showcases the changes in FDPs values of patients in the control and OGs over intervention time. According to Figure 1B, when the intervention time was on the 5th, 10th, and 15th day, the FDPs values of the CG patients were 26.392 ± 5.641, 24.610 ± 5.275, and 19.032 ± 5.101, respectively. The FDPs values of the OG patients were 22.892 ± 4.859, 15.627 ± 4.866, and 3.178 ± 4.125, respectively. Based on Figure 1, the D-D values and FDPs values of the CG and OG showed no significant SS before intervention (*P* > .05), but showed SSDs after 2 weeks of intervention (*P* < .05).

### 3.9. Comparison of nursing satisfaction among the participants

**Figure 2.**
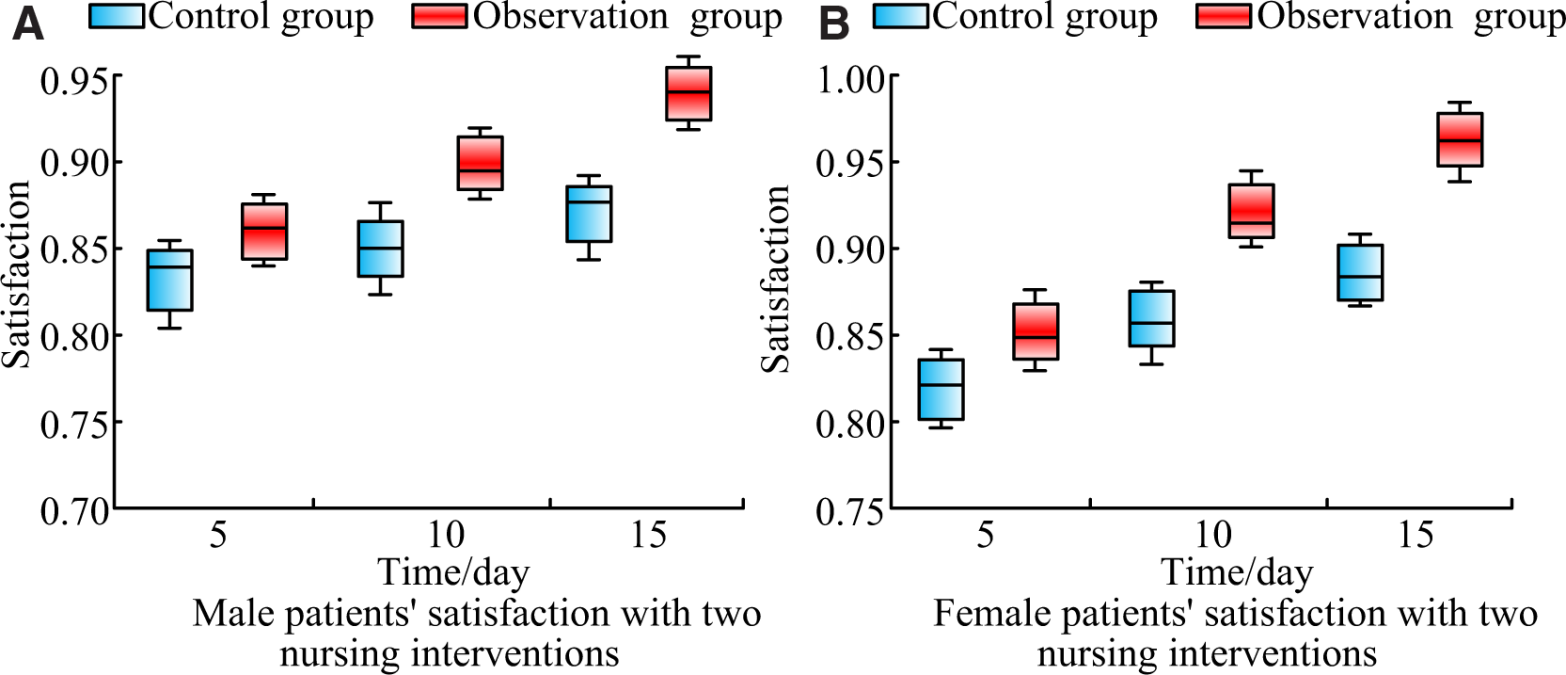
Satisfaction of male and female patients with the 2 nursing interventions.

This study divided all patients into male and female patient groups according to gender, and asked all patients to rate the 2 nursing interventions, with a maximum score of 1 point. The satisfaction of male and female patients with the 2 nursing interventions was obtained, as shown in Figure 2.

Figure 2 shows the satisfaction levels of male and female patients with general care and bundled care. The closer the satisfaction value is to 1, the more satisfied the patient is. Figure 2A shows the satisfaction of male patients with 2 nursing interventions. Figure 2A shows that male patients have different satisfaction levels with the 2 nursing interventions at different intervention time points. After 15 days of care, the satisfaction levels of male patients with the CG and OG are 0.865 and 0.942, respectively. Figure 2B showcases the satisfaction of female patients with 2 nursing interventions. Figure 2B showcases that the satisfaction of female patients with the 2 nursing interventions varies with the intervention time. After 15 days of care, the satisfaction of female patients with the CG and the OG is 0.878 and 0.965, respectively.

## 4. Discussion

In contemporary medical treatment, poststroke complications have an essential influence on the rehabilitation and LQ of patients, among which VTE is a particularly serious complication.^[13]^ VTE may not only exacerbate patient discomfort, but also lead to delayed recovery and even patient death. Especially for older stroke patients, as they age, their circulatory system slows down, and coupled with the lack of physical activity during postoperative recovery, they are prone to developing VTE.^[14]^ Therefore, for this group, taking effective and comprehensive nursing measures to control and prevent the occurrence of VTE is the key to improving clinical nursing effectiveness and patient LQ. Bundled care is a comprehensive medical care method that emphasizes patient-centered care, focusing on the overall needs of patients, including physical, psychological, and social aspects, to provide comprehensive nursing services.^[15,16]^ This study is for evaluating the effectiveness of bundled nursing strategies in preventing VTE in nonsurgical patients with cerebral hemorrhage. By comparing the incidence of VTE, coagulation function indicators, blood routine changes, LLM function, LQ, and patient satisfaction between the CG and the OG after receiving different nursing strategies, this study revealed the potential advantages of bundled nursing strategies in VTE prevention in nonsurgical patients with cerebral hemorrhage.

This study further confirmed the effectiveness of bundled nursing strategies in reducing the incidence of VTE. This was meaningful for improving the prognosis of nonsurgical patients with cerebral hemorrhage and reducing the burden on the medical system. Implementing this strategy for nonsurgical patients with cerebral hemorrhage can not only alleviate their physical pain, but also optimize their psychological state and social function, providing them with more comprehensive recovery support.^[17]^ The specific research results indicated that after receiving a bundled care strategy, the incidence of high-risk VTE in the OG decreased from 15% to 6%, while the incidence of high-risk VTE in the CG only decreased from 18% to 16%. The incidence of high-risk VTE in the OG was significantly below that in the CG, and after 2 weeks of intervention, there was a SSD in the number of VTE risk levels among the participants (*P* < .05). This finding was consistent with existing literature and emphasized the importance of preventive measures in reducing the incidence of VTE.^[18,19]^ The bundled nursing strategy provided patients with a comprehensive prevention plan by integrating various measures such as drug prevention, physical therapy, and nursing intervention. Compared to relying solely on a single intervention measure, this diversified approach was more adaptable to the individual needs of patients, thereby improving prevention effectiveness.^[20]^ In terms of cognitive indicators of lesions, the number of patients in the OG who had a healthy diet, mental health, no adverse drug reactions, and actively participated in exercise before intervention was 68, 53, 51, and 62, respectively. After 2 weeks of bundled nursing intervention, the number of patients with various indicators increased, ultimately becoming 98, 99, 96, and 95. In addition, there was a SSD (*P* < .05) in the comparison of lesion cognition among the participants after intervention. In terms of coagulation function indicators, the OG showed a decrease in the levels of D-dimer and FDPs after receiving bundled care. D-dimer and FDPs, as biomarkers for thrombus formation and dissolution, reflected changes in the balance between coagulation and fibrinolysis in the body. The decrease in D-Dimer and FDPs levels in the OG patients may indicate that the bundled nursing strategy effectively reduces the risk of thrombosis in patients, which may be attributed to the combined effects of drug prevention and physical therapy included in the nursing strategy. In addition, the OG showed an increase in APTT, TT, and PT values after receiving bundled care. This indicated that bundled nursing interventions are beneficial in reducing the incidence of VTE and improving the coagulation function of patients. In terms of blood routine indicators, the changes in PLT, WBC, and RBC counts of the OG patients also showed that bundled care may have a positive impact on the patient’s blood physiological status. After 2 weeks of bundled nursing intervention, the PLT, WBC, and RBC values of the OG were below those of the CG, and there was a SSD (*P* < .05) among the participants. In terms of LLM function, the OG showed a significant improvement in early rehabilitation training compared to the CG (*P* < .05), which may help reduce the risk of VTE caused by prolonged bed rest. This result highlighted the importance of early rehabilitation training in improving patient prognosis during the rehabilitation process of cerebral hemorrhage. Interventions such as promoting physical activity in patients may also decrease the risk of muscle atrophy and other complications caused by lack of activity. The improvement of LQ reflected the overall positive impact of bundled nursing strategies on the physical and mental health of patients. In terms of improving social and psychological functions, the OG showed significant progress (*P* < .05), which not only improved their LQ but may also promote better disease prognosis. Finally, in terms of patient satisfaction, the high satisfaction score of bundled care further confirmed the acceptance and effectiveness of this comprehensive nursing method. The increase in patient satisfaction with nursing may increase their willingness to follow medical advice, thereby improving overall treatment outcomes. Although this study has achieved certain results, there are still some limitations. For example, the limited sample size of the study, which only includes 200 patients, may limit the generalizability and statistical validity of the results. Future research should expand the sample size to obtain more representative and reliable conclusions.

## 5. Conclusion

In summary, the bundled care strategy offers a comprehensive treatment plan for patients with nonsurgical cerebral hemorrhage by integrating various prevention and intervention measures. It effectively reduces the risk of VTE, improves coagulation function and blood routine indicators, enhances lower limb motor (LLM) function, and improves quality of life, gaining high patient recognition. Compared to ordinary nursing, bundled care better improves coagulation status and routine blood indexes and helps prevent venous thromboembolism in these patients.

## Author contributions

**Conceptualization:** Lu Hongfang, Tian Yangyang, Sun Na.

**Data curation:** Lu Hongfang, Tian Yangyang, Zhao Lijuan, Sun Na.

**Formal analysis:** Lu Hongfang, Tian Yangyang, Zhao Lijuan, Sun Na.

**Funding acquisition:** Lu Hongfang, Tian Yangyang.

**Investigation:** Lu Hongfang, Tian Yangyang, Zhao Lijuan.

**Methodology:** Lu Hongfang, Tian Yangyang, Zhao Lijuan.

**Supervision:** Zhao Lijuan, Sun Na.

**Visualization:** Zhao Lijuan.

**Writing – original draft:** Lu Hongfang.

**Writing – review & editing:** Lu Hongfang, Sun Na.
